# Physiological Insights into Enhanced Epsilon-Poly-l-Lysine Production Induced by Extract Supplement from Heterogeneous *Streptomyces* Strain

**DOI:** 10.3390/microorganisms13081868

**Published:** 2025-08-10

**Authors:** Siyu Tong, Chen Zhang, Zhanyang Zhang, Huawei Zeng, Bingyue Xin, Mingtao Zhao, Deyin Zhao, Xin Zeng, Fei Zhang

**Affiliations:** 1 Anhui Province Key Laboratory of Pollutant Sensitive Materials and Environmental Remediation, Huaibei Normal University, Huaibei 235000, China; t13637219368@163.com (S.T.); zc199910162023@163.com (C.Z.); zyzhang123123@163.com (Z.Z.); huaweizeng@163.com (H.Z.); xinbingyuex@163.com (B.X.); chnuzmt2025@163.com (M.Z.); zdy1178363152@163.com (D.Z.); 2 College of Life Sciences, Huaibei Normal University, Huaibei 235000, China

**Keywords:** interspecies signaling, *Streptomyces albulus*, *Streptomyces gilvosporeus*, ε-poly-l-lysine, systemic biological analysis

## Abstract

Epsilon-poly-l-lysine (ε-PL) is a potent antimicrobial agent, but strategies to enhance its biosynthesis remain limited due to insufficient understanding of its physiological regulation. This study explores the interaction between *Streptomyces albulus* and heterogeneous microbial extracts, with a focus on actinomycete-derived signals. The *S. gilvosporeus* extract induces the highest ε-PL production (3.4 g/L), exceeding the control by 2.6-fold and outperforming *B. cinerea* by 1.8-fold. Multi-omics analyses combined with morphological and biochemical profiling reveal that the induced state is characterized by intensified central carbon flux, enhanced lipid turnover, elevated respiratory activity, and cofactor regeneration, alongside suppression of competing secondary pathways. Morphological alterations, including denser mycelial aggregation and compact colony structures, accompany these metabolic shifts. Compared to *B. cinerea*, *S. gilvosporeus* elicits more pronounced stress adaptation and metabolic reprogramming in *S. albulus*. These findings suggest that interspecies interactions can activate intrinsic aggression resistance mechanisms, thereby driving ε-PL biosynthesis through a previously unrecognized physiological route.

## 1. Introduction

ε-PL is an amino acid polymer composed of 25–35 L-lysine residues linked through dehydration condensation between the α-carboxyl and ε-amino groups [1]. As a biodegradable polymer with broad-spectrum antimicrobial activity, excellent water solubility, a wide pH tolerance range, and high biological safety, ε-PL has been widely adopted as a safe and environmentally friendly bioactive compound across the food, pharmaceutical, and personal care industries [2]. In the food sector, ε-PL effectively inhibits *Penicillium expansum* on apples and *Botrytis cinerea* on various fruits and vegetables, thereby reducing spoilage and extending shelf life [3,4]. Its combination with tea polyphenols enhances the microbial stability and sensory quality of meat products [5,6], while synergy with licoricidin demonstrates potent anti-biofilm activity [7]. In pharmaceuticals, the cationic nature and biofilm-penetrating capacity of ε-PL enable its application in drug and gene delivery systems [8], including micellar formulations for glioma gene therapy [9], adhesive materials for wound closure [10], and self-degradable medical glues [11]. In the personal care field, ε-PL combined with FP and domipramine exhibits strong antimicrobial and anti-halitosis effects, as confirmed in clinical trials [12]. Despite its promising functionalities, the industrial and commercial application of ε-PL is constrained by its high production cost (approximately USD 200/kg). Consequently, improving fermentation efficiency to reduce manufacturing costs remains a major focus of both academic and industrial research.

ε-PL is a secondary metabolite primarily synthesized by *Streptomyces albulus* under acidic conditions. The efficient biosynthesis of ε-PL necessitates the concurrent fulfillment of three key factors: highly active Pls (ε-PL synthetase, a member of the non-ribosomal peptide synthetase (NRPS) family), elevated intracellular ATP levels, and rapid provision of lysine precursors. To enhance the yield and efficiency of ε-PL fermentation, research efforts have focused on the following three aspects: (i) construction of efficient ε-PL-producing strains through random mutagenesis and directed evolution, including mutation screening [13], genome shuffling [14], and ribosome engineering [15]; (ii) metabolic enhancement of the key biosynthetic pathways involved in ε-PL synthesis using advanced genetic engineering methods [16,17]; and (iii) optimization of media formulations and precise control of culture parameters [18,19,20,21,22].

However, current strategies primarily follow the paradigm of “meeting the substrate requirements for ε-PL synthesis at either macroscopic or microscopic levels”, but they often overlook a fundamental question: “Why do ε-PL-producing strains actively synthesize this product?” In other words, “What is the physiological significance of ε-PL synthesis for the producing strains?” Compared to humans, the producing strains inherently possess the mechanisms that govern why and how they synthesize ε-PL efficiently. Therefore, only by comprehending the physiological significance of ε-PL synthesis for the producing strains and stimulating their intrinsic driving force can we achieve comprehensive metabolic enhancement.

Given that an acidic shock triggers excessive production of ε-PL [23,24,25,26,27], the potential physiological function of ε-PL in producing strains—to neutralize the surrounding acidic environment through alkaloid production—has attracted growing attention. However, other than the acidic stimulus, no other intrinsic driving forces have been identified over the past decade. Interestingly, ε-PL-producing strains predominantly belong to the actinomycetes, which exhibit a slower growth rate compared to most bacteria and have less efficient mycelium and spore dispersal capabilities than molds. This may indicate that ε-PL, a broad-spectrum bacteriostatic agent, enables these *Streptomyces* species to compete with other microorganisms for territory and nutrients. We hypothesize that the intrinsic drive to produce ε-PL stems from the need to resist invasion by competing microorganisms.

In this study, we found that certain other microorganisms could significantly enhance ε-PL production, particularly in *Streptomyces* species. To understand the underlying mechanisms, we conducted systematic physiological analyses, examining intracellular messengers, mycelial morphology, gene transcription, key enzymes in ε-PL production, and intracellular precursor pools.

## 2. Materials and Methods

### 2.1. Microorganisms and Culture Media

The strains utilized in this study were acquired from the China Center of Industrial Culture Collection (CICC), China General Microbiological Culture Collection Center (CGMCC), and American Type Culture Collection (ATCC). Detailed information is provided in Table 1. For the pre-activation of all microorganisms, an agar slant medium was used, consisting of 10 g/L glucose, 5 g/L yeast extract powder, 5 g/L peptone, and 20 g/L agar, with the initial pH adjusted to 7.5 using 2 M NaOH. This same medium was employed for spore preparation of the ε-PL-producing strain *Streptomyces albulus* IFO14147. A modified LB medium was used for bacterial cultures, containing 10 g/L glucose, 10 g/L tryptone, 5 g/L yeast extract powder, 0.5 g/L K_2_HPO_4_, 0.5 g/L MgSO_4_·7H_2_O, 0.04 g/L ZnSO_4_·7H_2_O, and 0.03 g/L FeSO_4_·7H_2_O, with the initial pH adjusted to 7.0 using 2 M NaOH. A modified YPD medium was used for fungal growth, composed of 40 g/L glucose, 10 g/L yeast extract powder, 20 g/L peptone, 0.5 g/L K_2_HPO_4_, 0.5 g/L MgSO_4_·7H_2_O, 0.04 g/L ZnSO_4_·7H_2_O, and 0.03 g/L FeSO_4_·7H_2_O, with the initial pH adjusted to 6.5 using 2 M NaOH. For the growth of *Streptomyces* species, a GYS medium was applied, which contains 60 g/L glucose, 10 g/L yeast extract, 20 g/L soy peptone, 5 g/L NaCl, and 5 g/L MgSO_4_·7H_2_O, with the initial pH adjusted to 7.5 using 6 M NaOH. Medium 3G (M3G) was specifically designed to support seed cultivation and formal ε-PL production, comprising 60 g/L glucose, 5 g/L yeast extract, 10 g/L (NH_4_)_2_SO_4_, 1.36 g/L KH_2_PO_4_, 0.8 g/L K_2_HPO_4_, 0.5 g/L MgSO_4_·7H_2_O, 0.04 g/L ZnSO_4_·7H_2_O, and 0.03 g/L FeSO_4_·7H_2_O, with the initial pH adjusted to 6.8 using 2 M NaOH. To prevent the Maillard reaction, glucose in all media was sterilized separately during autoclave treatment at 121 °C for 20 min.

### 2.2. ε-PL Production After the Addition of Other Microorganisms

The activation of strains was conducted on agar slant medium at 30 °C for 2–10 days, with the exception of *B. cinerea*, which was activated at 24 °C for 3–5 days. For the preparation of crude microbial extracts, inducing bacteria listed in Table 1 were cultured in a modified LB medium at 28 °C and 200 rpm for 15–24 h in a rotary shaker; inducing fungi from Table 1 were cultured in a modified YPD medium at 28 °C and 180 rpm for 2–4 days under the same conditions; *Streptomyces* species from Table 1 were incubated in a GYS medium at 28 °C and 200 rpm for 1–3 days in a rotary shaker. After cultivation, the broth was centrifuged at 6000× *g* for 10 min. The sediments (biomass) were used as the inducing microorganisms after sterilization at 115 °C for 15 min. The ε-PL-producing strain, *S. albulus* IFO 14147, was inoculated on an agar slant medium and incubated at 30 °C for 9 days until spore formation. For the seed pre-culture, 80 mL of M3G medium (in 500 mL flasks) was inoculated with one loop of spores (approximately 5 × 10^5^ spores) and incubated at 200 rpm and 30 °C in a rotary shaker for 24 h. To initiate ε-PL production, 8% of the seed broth was transferred into 30 mL of M3G (in 250 mL flasks). The flasks were incubated at 200 rpm and 30 °C for 16 h, during which the pH naturally dropped to 4.0. Subsequently, a mixture of the prepared crude microbial extracts (harvested from former cultures) and citrate buffer (a final concentration in the broth of 10 g/L, pH 4.0 adjusted using 6 M NaOH) was added. The total culture time was set to 48 h, after which the final ε-PL titer in the broth was measured.

### 2.3. Primary Extraction of Microbial Signal Mixture and Its Influence on ε-PL Production

Microbial extract mixtures were obtained from the biomass of selected fungi and actinomycetes that exhibited a positive effect on ε-PL production [28]. The fermentation broth was centrifuged at 5000× *g* for 10 min, and the resulting sediment was washed twice with deionized water to isolate the biomass. The biomass was then suspended in a 75% ethanol solution, ground with quartz sand, and centrifuged again at 5000× *g* for 20 min to separate the supernatant (S_n_) and sediment (S_d_). Ethanol was removed from the extract by evaporation, and the remaining solution was divided into four equal parts. Each part was sequentially extracted using ethyl acetate, chloroform, butyl alcohol, and petroleum ether. The organic phases were collected using a separating funnel and evaporated to remove the organic solvents. The resulting extracts were designated as fraction E (ethyl acetate extract), fraction C (chloroform extract), fraction B (butyl alcohol extract), and fraction P (petroleum ether extract). The effects of these crude microbial extracts on ε-PL production were evaluated via exogenous addition at 24 h in 250 mL flasks.

### 2.4. ε-PL Production After the Addition of S. gilvosporeus Extracts in a 5 L Fermenter

The impact of the *S. gilvosporeus* extracts on ε-PL production was evaluated through fed-batch cultures conducted in a 5 L jar fermenter (Baoxing Corp., Shanghai, China) with a working volume of 3 L. Profiles of fed-batch cultures without *S. gilvosporeus* extract addition (CK, control group) were compared to those with the extract addition (EG, experimental group). In the experimental group, crude extracts of S. gilvosporeus were added at a concentration of 36 g wet cells/L. The fermentation parameters were set as described previously [29]. The fermenter was equipped with two turbine agitators and automated control systems for temperature, dissolved oxygen (DO), and broth pH. Cultures were initiated after inoculation of the seed broth at an 8% ratio and maintained at 30 °C. Agitation rates ranged from 200 to 800 rpm, and aeration was set at 1.0 vvm to maintain DO levels at 30% throughout the culture period. When the broth pH naturally dropped to 4.0, 12.5% (*w*/*v*) NH_3_·H_2_O was automatically added to maintain a constant pH of 4.0. When the residual glucose concentration decreased to below 10 g/L, 70% of the sterilized glucose was automatically supplemented to maintain a concentration range of 5–15 g/L. Fed-batch cultures were terminated at 60 h, after which the impact of microbial extracts might be affected by multiple reasons. Broth samples were collected at specific time points for analysis of ε-PL titer and the DCW.

### 2.5. Impacts of S. gilvosporeus Extracts on the Morphology of S. albulus IFO 14147

The effects of *S. gilvosporeus* extracts on the morphology of *S. albulus* IFO 14,147 were assessed through macroscopic and microscopic observations. Macroscopically, the colony morphology on *Petri* plates was evaluated by comparing plates with and without the addition of fraction E extracts at a final concentration of 36 g wet cells/L. Microscopically, mycelial morphology in submerged cultures was examined using scanning electron microscopy (SEM). For macroscopic analysis, *Petri* plates containing agar slant medium supplemented with fraction E were inoculated with diluted spore suspensions of *S. albulus* IFO 14147. Colony morphology was observed at 3 and 6 days post-inoculation and compared to control plates without added extracts. For microscopic analysis, samples from submerged cultures were collected at 45 h with the addition of extracts at 24 h. These samples were fixed with glutaraldehyde, buffered with phosphate buffer, dehydrated with ethanol, and then observed using SEM (Sigma 300, ZEISS, Oberkochen, Germany). Cell viability was assessed using a CTC (5-cyano-2, 3-ditolyl tetrazolium chloride) Rapid Staining Kit (Dojindo Laboratories, Kumamoto, Japan), as previously described [29], and images of mycelial morphology after CTC staining were captured using a fluorescence microscope (BX51, Olympus, Tokyo, Japan).

### 2.6. RNA Extraction and Transcriptomic Profiling

The most pronounced transcriptional divergence between CK and EG cultures occurred between 36 and 60 h. Thus, 36 h mycelial pellets were selected for RNA-seq analysis. Triplicate cultures were harvested at this time point, centrifuged (7000× *g*, 1 min), washed, flash-frozen in liquid nitrogen, and stored at −80 °C. Total RNA was extracted using established protocols [30], and its quality was assessed via agarose gel electrophoresis and an Agilent (Santa Clara, CA, USA) 2100 bioanalyzer to ensure integrity and purity. For library construction, mRNA was isolated from non-coding rRNA, reverse transcribed into cDNA, and processed with end-repair and adapter ligation. Sequencing was performed on the Illumina platform to yield 150bp paired-end reads, followed by alignment to the *Streptomyces albulus* IFO14147 genome (CP104098.1) [31]. Differentially expressed genes (DEGs) were identified with Log_2_^(EG/CK)^ > 1 (*p* < 0.05) through DESeq2, and functional enrichment analysis of these DEGs was conducted using GO and KEGG annotations to elucidate the underlying mechanisms of the fermentation enhancement derived from the addition of *Streptomyces* extract. Priority was given to transcriptional alterations in lipid catabolism and energy metabolism pathways for functional analysis.

### 2.7. Transcriptional Performances of Key Genes in ε-PL Biosynthesis

To explore the transcriptional levels of ε-PL biosynthesis-related genes, qRT-PCR was utilized to analyze the key genes using the samples collected at 42 h from the CK and EG fed-batch cultures in a fermenter. The total RNA extraction from *S. albulus* IFO 14,147 was carried out following the protocol reported previously [29]. The transcription of genes *N1H47_11860, N1H47_11275, N1H47_14640, N1H47_21215*, and *N1H47_34205* was quantified via real-time fluorescent PCR (ABI Stepone plus, Applied Biosystems, USA) with SG Fast qPCR Master Mix (High Rox, Bio Basic Inc., Canada). The primer sequences for these genes are detailed in Table S4.

### 2.8. Activity Assay of Key Enzymes and Electron Transport System in ε-PL Biosynthesis

For the measurement of key enzymes and ETS, the broth samples were withdrawn from the fermenter at 36 h, 42 h, 48 h, 54 h, and 60 h. The cell extract preparation and activity assay of phosphoenolpyruvate carboxylase, pyruvate kinase, citrate synthase, aspartate kinase, and ETS were conducted following the procedures described previously [31,32,33,34,35].

### 2.9. Metabolomic Profiling via UPLC-ESI-MS/MS

Metabolic variations between CK and EG were analyzed using ultra-performance liquid chromatography coupled with electrospray ionization tandem mass spectrometry (UPLC-ESI-MS/MS). Cell samples were harvested from triplicate 42 h CK and EG cultures. Intracellular metabolites were quantified on a Vanquish UHPLC system (Thermo Fisher Scientific, Waltham, MA, USA) equipped with an ACQUITY UPLC^®^ HSS T3 column (150 × 2.1 mm, 1.8 μm; Waters, Milford, MA, USA) and a Q Exactive mass spectrometer (Thermo Fisher Scientific, Waltham, MA, USA) employing ESI ionization, following established protocols [30]. Mass Profiler Professional 13.0 (Agilent, Santa Clara, CA, USA) facilitated metabolite profile analysis, with compound identification based on retention time and mass spectra using a laboratory-developed database. Multivariate statistical analyses, including principal component analysis (PCA) and orthogonal partial least squares discriminant analysis (OPLS-DA), were performed using SIMCA 14.1 (Umetrics AB, Umeå, Sweden) to resolve inter-group metabolic disparities [36]. The Z-score was calculated to visualize the metabolic profiling.

### 2.10. Analytical Methods

After centrifugation of the fermentation broth at 5000× *g* for 10 min, the clarified supernatant was subjected to quantitative analysis of ε-PL production and residual glucose content. Simultaneously, the pelleted biomass was collected for DCW determination. All analytical procedures, including ε-PL quantification, glucose consumption monitoring, and DCW measurement, were performed according to the standardized methods established in our prior research [29]. The concentration of ε-poly-L-lysine (ε-PL) was determined by diluting the fermentation supernatant with 0.7 mM sodium phosphate buffer (pH 7.0). A 2 mL aliquot of this diluted sample was mixed with 2 mL of 1 mM methyl orange solution. The mixture was allowed to react at 30 °C for 30 min, followed by centrifugation at 4500× *g* for 15 min. The resulting supernatant was diluted 20-fold with the same phosphate buffer, and its absorbance was measured at 465 nm using a spectrophotometer. The concentration of ε-PL was determined by referring to a previously established standard curve depicting the correlation between A465 and the concentration of ε-PL. The determination of dried cell weight (DCW) was accomplished using a gravimetric method that relied on the weight difference in filter paper. A 10 mL sample of the fermentation broth was withdrawn from the 5 L fermenter and centrifuged at 4500× *g* for 10 min. The resulting biomass pellet was washed twice with distilled water and then filtered through pre-weighed filter paper (Φ 7 cm, medium speed, SCRC) that had been previously dried at 105 °C. Subsequently, the filter paper with the biomass was dried at 105 °C until a constant weight was achieved and then reweighed. The DCW was calculated by measuring the weight difference before and after filtration.

### 2.11. Calculations

The average values of the specific cell growth rate and the specific ε-PL formation rate were calculated in the following manner:

(1) $$\mathrm{Average}\;\mathrm{specific}\;\mathrm{cell}\;\mathrm{growth}\;\mathrm{rate}=\;\frac{1}{\frac{1}{2}\;[{c\left(X\right)}_{t_{2}}+{c\left(X\right)}_{t_{1}}]}\times \frac{{c(X)}_{t_{2}}-{c(X)}_{t_{1}}}{t_{2}-t_{1}}$$

(2) $$\mathrm{Average}\;\mathrm{specific}\;\varepsilon \text{-}\mathrm{PL}\;\mathrm{formation}\;\mathrm{rate}=\frac{1}{\frac{1}{2}\;[{c\left(X\right)}_{t_{2}}+{c\left(X\right)}_{t_{1}}]}\;\times \frac{{c(P)}_{t_{2}}-{c(P)}_{t_{1}}}{t_{2}-t_{1}}$$

The above parameters are mean values over a time range of 12 h, where *t*, *c*(*X*), and *c*(*P*) represent the culture time (h), DCW (g/L), and ε-PL titer (g/L) at the *t* h, respectively.

### 2.12. Statistical Processing

All experimental data in this work were derived from three or more independent replicates. Data visualization was executed using GraphPad Prism 9.0 (GraphPad Software, San Diego, CA, USA), while statistical evaluations, including significance testing, were conducted through SPSS 23.0 (IBM Corp, Armonk, NY, USA). The quantitative results are expressed as mean values with standard deviations (mean ± SD, *n* ≥ 3), with *p*-values below 0.05 considered statistically significant. For all statistically significant comparisons, the corresponding effect sizes (Cohen’s d > 0.8) indicated large effect magnitudes.

## 3. Results

### 3.1. ε-PL Production After the Addition of Heterogeneous Microorganisms’ Extracts

**Figure 1:**
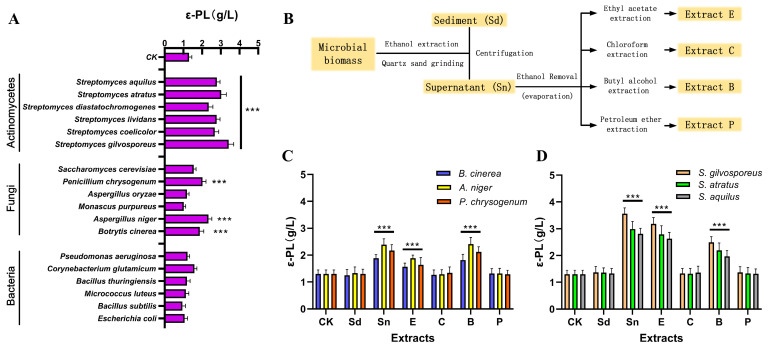
Inducing effect of microorganisms on ε-PL production. (**A**) ε-PL production after addition of heterogeneous microorganisms (dead cells); (**B**) primary extraction procedure of microbial signal mixture; (**C**) effects of fungi extracts on ε-PL production; (**D**) influences of actinomycetes extracts on ε-PL production. Mean ± SD, *n* ≥ 3, biological replicates. *** indicates *p* < 0.001.

The effect of extracts from different microorganisms (bacteria, fungi, and actinomycetes) on ε-PL production by *S. albulus* was systematically evaluated (Figure 1A). Among these, extracts from several *Streptomyces* species proved most effective, yielding a more than 1.8-fold increase in ε-PL titers compared to other microbial groups. Certain fungal species, including *B. cinerea*, *A. niger*, and *P. chrysogenum*, also elevated ε-PL titers. Conversely, none of the tested bacterial strains improved ε-PL biosynthesis. Among the fungi, *A. niger* was the top performer, yielding 2.3 g/L, while *S. gilvosporeus* was the most effective actinomycete, producing 3.4 g/L. Thus, extracts from various *Streptomyces* species are particularly effective at enhancing ε-PL biosynthesis, with those from *S. gilvosporeus* showing the most pronounced effect. To better understand the impact of microbial signals on ε-PL biosynthesis, we extracted biomass from strains (Figure 1B) that positively influenced ε-PL production and assessed their effects on ε-PL synthesis (Figure 1C,D). The data suggested that potential microbial signals from fungal and actinomycete biomass were soluble in 75% ethanol (Sn). Furthermore, these potential microbial signals were detectable in ethyl acetate (extract E) and butyl alcohol (extract B), but not in chloroform (extract C) or petroleum ether (extract P). These findings suggest that the functional signals from fungi and actinomycetes are likely small molecules. Notably, intriguing differences were observed between extracts from fungi and those from actinomycetes. For fungi, higher ε-PL titers were detected in extract B, while extract E yielded greater ε-PL concentrations for actinomycetes (Figure 1C,D). These observations underscore the structural distinctions in functional signals between fungi and actinomycetes, indicating that fungal signals exhibit higher polarity compared to those of actinomycetes.

### 3.2. ε-PL Fermentation Profile After Addition of S. gilvosporeus Biomass Extract

**Figure 2:**
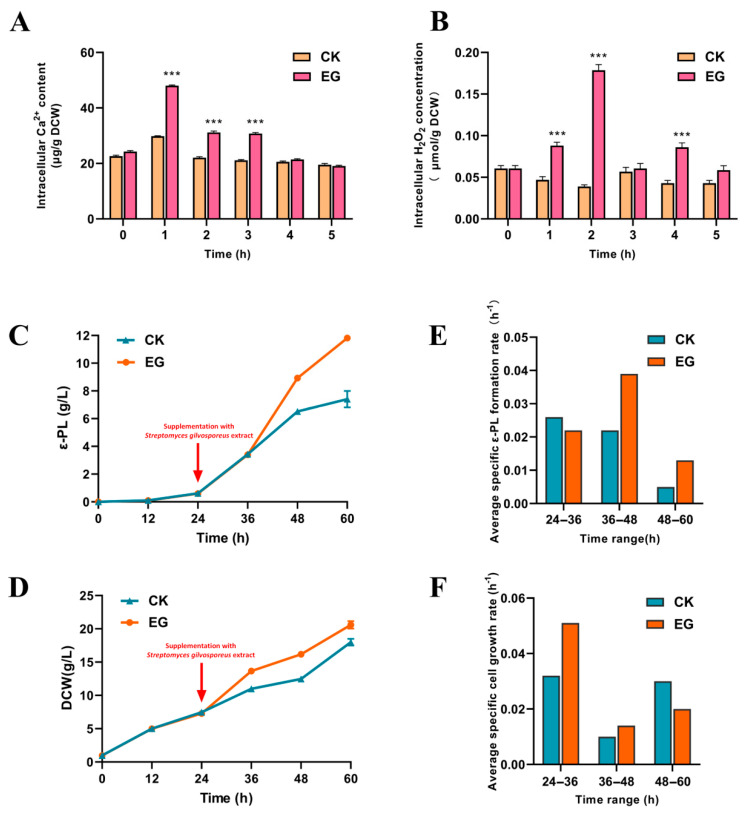
Changes in intracellular secondary messengers (H_2_O_2_ and Ca^2+^) and profiles of fermentation parameters in ε-PL production upon addition of *S. gilvosporeus* extracts. (**A**) Intracellular Ca^2+^ content upon addition of *S. gilvosporeus* extracts. (**B**) Intracellular H_2_O_2_ content upon the addition of *S. gilvosporeus* extracts. (**C**) Profiles of ε-PL concentrations in cultures with (EG) or without (CK) addition of *S. gilvosporeus* extracts. (**D**) Profiles of dried cell weight (DCW) in cultures with (EG) or without (CK) addition of *S. gilvosporeus* extracts. (**E**) Average specific ε-PL formation rate in cultures with (EG) or without (CK) addition of *S. gilvosporeus* extracts. (**F**) Average specific cell growth rate in cultures with (EG) or without (CK) addition of *S. gilvosporeus* extracts. Mean ± SD, *n* ≥ 3, technical replicates. *** indicates *p* < 0.001.

The *S. gilvosporeus* extracts induced a marked increase in intracellular H_2_O_2_ and Ca^2+^ levels over the subsequent 5-h period (Figure 2A,B), potentially triggering extensive physiological modifications via ROS- and Ca^2+^-dependent regulatory pathways. Furthermore, the experimental group (EG) treated with the *S. gilvosporeus* extracts exhibited higher ε-PL concentrations (Figure 2C) and increased dried cell weight (DCW) (Figure 2D) compared to the control group (CK). Additionally, Figure 2E,F shows a sequential enhancement in cell growth between 24 and 36 h of cultivation, followed by an increase in ε-PL biosynthesis from 36 to 60 h. The significant improvement in ε-PL biosynthesis upon stimulation via the *S. gilvosporeus* extracts warrants further investigation into the underlying physiological mechanisms.

### 3.3. Cell Morphology of S. albulus After S. gilvosporeus Extract Addition

**Figure 3:**
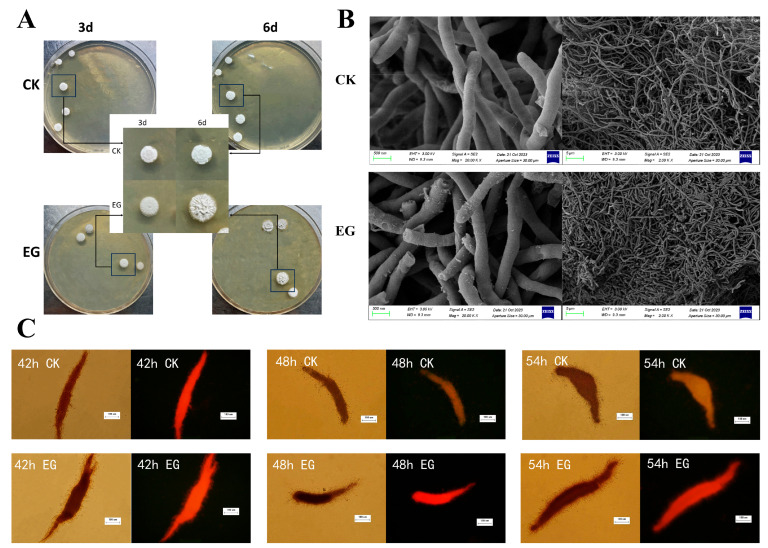
Morphological comparison between cultures with (EG) or without (CK) addition of *S. gilvosporeus* extracts. (**A**) Morphology of *Petri* plate colony in agar medium. (**B**) Morphology of mycelia and mycelial pellets in submerged culture with M3G medium via SEM. (**C**) CTC staining images of mycelial pellets in submerged culture with M3G medium at 42 h, 48 h, and 54 h.

As shown in Figure 3A, macroscopic colonies exposed to the *S. gilvosporeus* extracts were significantly larger and showed a pronounced tendency to aggregate centrally, an effect that was particularly evident by day 6. Similarly, in liquid culture, the extracts induced denser and more organized microscopic mycelial structures with increased excretion (Figure 3B). The CTC assay confirmed this enhanced metabolic state, showing that the extracts substantially increased mycelial respiratory activity over an extended period (42–54 h) (Figure 3C).

### 3.4. Transcriptome Performance After S. gilvosporeus Extract Addition

**Figure 4:**
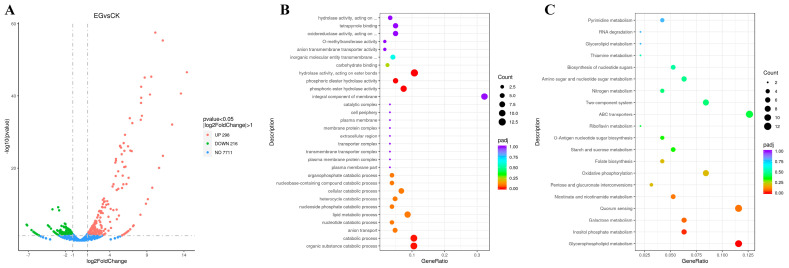
Transcriptome analysis of cell samples from cultures with (EG) or without (CK) *S. gilvosporeus* extract addition. (**A**) Volcano diagrams revealing a comparison of the number of differentially expressed genes in cultures between EG vs. CK. (**B**) The top 30 significantly enriched (*padj* < 0.05) GO terms in “EG vs. CK” comparison. (**C**) The top 30 significantly enriched (*padj* < 0.05) KEGG pathways in “EG vs. CK” comparison. Mean ± SD, *n* ≥ 3, technical replicates.

As shown in Figure 4A, the *S. gilvosporeus* extracts induced a significant transcriptional response, upregulating 296 genes and downregulating 216 genes. Many of these changes were substantial (Log_2_^FoldChange^ > 4, −log_10_^(*p*-value)^ > 5), suggesting that the extracts activated numerous previously dormant genes. GO clustering analysis (Figure 4B) of the top 30 differentially expressed genes indicated that the *S. gilvosporeus* extracts activated global catabolic pathways, particularly those involving the hydrolysis of phosphoric esters and diesters. Specifically, three of the seven genes encoding phospholipase were significantly upregulated (Table 2). KEGG cluster analysis (Figure 4C) showed that the top 30 significantly differentially expressed genes were concentrated in pathways for signal transduction (e.g., quorum sensing and two-component systems), ABC transporters, and the metabolism of nicotinate, nicotinamide, and glycerophospholipids, among other processes. Notably, all subunits (A–F, H) of NADH-quinone oxidoreductase in the EG displayed significantly higher transcription levels compared to the CK, although F0F1 ATP synthase in the EG did not show substantial transcriptional changes (Table 3). These findings suggest that *S. albulus* may be regulating itself to boost energy production via intensive cell respiration, primarily through lipid hydrolysis and oxidation.

### 3.5. Transcriptional and Enzymatic Performance of Key Genes in ε-PL Biosynthesis After S. gilvosporeus Extract Addition

**Figure 5:**
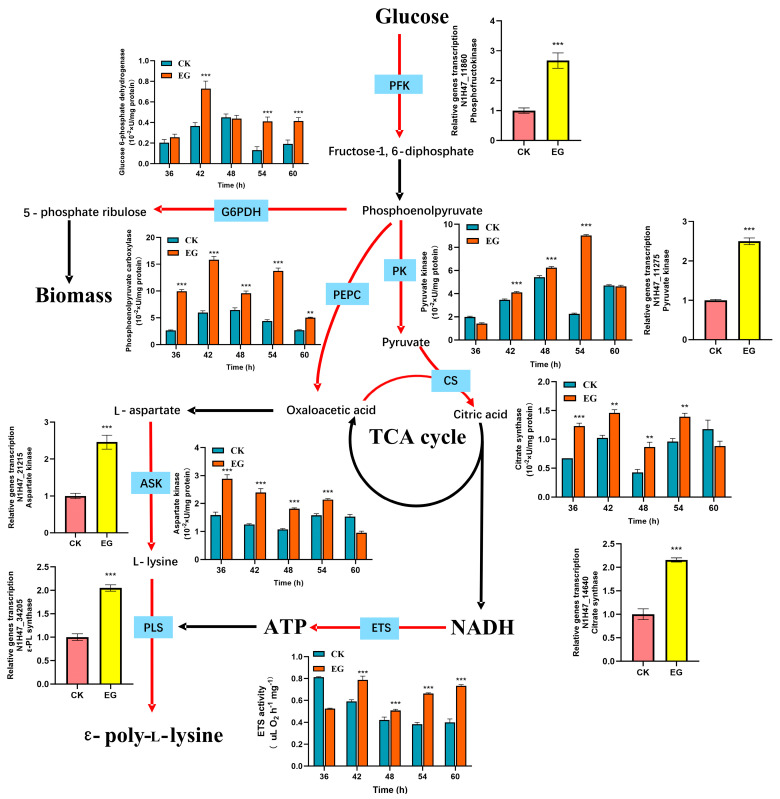
Effects of *S. gilvosporeus* extract addition (24 h) on the transcription of key genes, intracellular activities of key enzymes in ε-PL biosynthesis metabolism. The gene transcription in CK (without *S. gilvosporeus* extract addition) was set as 1 (dot lines), and those genes’ relative transcription in EG (with *S. gilvosporeus* extract addition) was calculated based on the levels of CK. Mean ± SD, *n* ≥ 3, technical replicates. *** indicates *p* < 0.001, ** indicates *p* < 0.001.

To elucidate the impact of *S. gilvosporeus* extracts on ε-PL biosynthesis, a comprehensive analysis integrating gene transcription and enzyme activity was conducted, as shown in Figure 5. The extracts enhanced the metabolic pathways specific to ε-PL production. Notably, increased activity of glucose-6-phosphate dehydrogenase was observed in the EG during the 42–60 h period. This pathway is crucial for NADPH generation, a key cofactor that facilitates both cell growth and ε-PL synthesis. The extract also significantly upregulated the transcription of key genes in the Embden–Meyerhof–Parnas (EMP) pathway, including *N1H47_11860* (encoding phosphofructokinase) and *N1H47_11275* (encoding pyruvate kinase). Phosphofructokinase is a key regulator of glycolytic flux, while pyruvate kinase links glycolysis to the TCA cycle by catalyzing its final step. This metabolic enhancement was confirmed by higher pyruvate kinase activity in the EG (42–54 h). The increased glycolytic flux was subsequently channeled into the TCA cycle, as evidenced by elevated transcription of the citrate synthase gene (*N1H47_14640*) and increased activity of the enzyme itself. Since citrate synthase catalyzes the first committed step of the TCA cycle—the conversion of oxaloacetate and acetyl-CoA into citrate—this upregulation directly facilitated greater NADH production. Concurrently, the activities of the electron transport system (ETS) were elevated in the EG during the 42–60 h period, indicating efficient ATP production from NADH. The ETS is crucial for oxidative phosphorylation, where NADH is used to drive ATP synthesis, supplying the energy necessary for ε-PL biosynthesis. Notably, phosphoenolpyruvate carboxykinase, a crucial enzyme for replenishing intermediates in the TCA cycle, showed significant activity improvement in the EG throughout the entire time range (36–60 h), ensuring timely replenishment of TCA cycle metabolite pools. By converting oxaloacetate into phosphoenolpyruvate, this enzyme facilitates the gluconeogenesis pathway, supporting the supply of carbon skeletons. This facilitated the efficient conversion of carbon skeletons (oxaloacetate) into L-lysine via aspartate kinase and subsequently into ε-PL through ε-PL synthase. In the EG, the upregulated transcription of *N1H47_21215* (aspartate kinase) and *N1H47_34205* (ε-PL synthase), along with higher aspartate kinase activity during the 36–54 h period, ensured the high efficiency of these metabolic processes. The overall metabolic enhancement induced by *S. gilvosporeus* extracts laid a solid foundation for rapid cell growth and improved ε-PL production.

### 3.6. Effect of S. gilvosporeus Extracts on the Metabolic Pools of Intermediates in Pathways for ε-PL Biosynthesis

**Figure 6:**
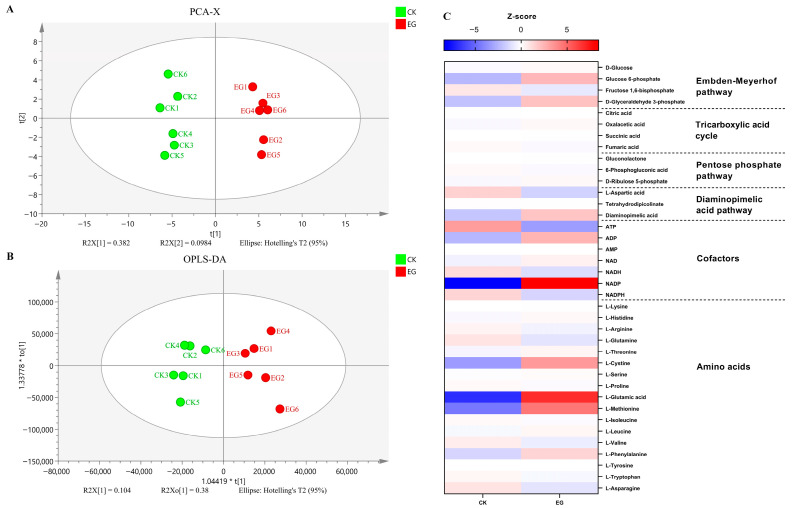
Metabolome analysis of cell samples from cultures with (EG) or without (CK) *S. gilvosporeus* extract supplement. (**A**) PCA score plot of CK and EG. (**B**) OPLS-DA score plot of “EG vs. CK” comparison group. (**C**) Heat map for Z-score of important intermediate metabolites in ε-PL biosynthesis metabolism. Relative content of significantly different metabolites calculated according to the OPLS-DA model (*p* < 0.05). Mean ± SD, *n* ≥ 6, technical replicates.

Principal component analysis (PCA) and Orthogonal Projections to Latent Structures Discriminant Analysis (OPLS-DA) were employed to assess the data quality of the non-targeted metabolomic assay using a UPLC-ESI-MS system. The score plot (Figure 6A) demonstrates a distinct separation between the EG and the CK. The two principal components derived from the PCA model accounted for 38.2% of the total variance. Furthermore, the OPLS-DA score scatter plots (Figure 6B) revealed a clear distinction between EG and CK. As illustrated in Figure 6C and Table S1, the *S. gilvosporeus* extracts (EGs) induced significant metabolic changes in the EMP pathway, the diaminopimelic acid pathway (DAP), and the intracellular composition of energy cofactors and amino acids. Within the EMP pathway, the concentrations of most metabolites increased following extract addition, exemplified by glucose 6-phosphate and glyceraldehyde 3-phosphate. The observed decrease in fructose-1,6-diphosphate is likely attributable to its rapid consumption for downstream metabolite synthesis. This metabolic reprogramming of the EMP pathway under EG conditions probably enhanced the supply of carbon skeletons for subsequent energy metabolism and product synthesis. In contrast, the metabolite pools in the TCA cycle and DAP changed minimally in response to the extract. A significant shift also occurred in the energy cofactor pools: low-energy molecules (ADP, NAD^+^) increased, while their high-energy counterparts (ATP, NADH) decreased. This trend was also observed for NADP^+^ and its reduced form, NADPH.

Interestingly, the extracts from *S. gilvosporeus* not only enhanced ε-PL production but also significantly modulated the composition of other secondary metabolites (Table S2). Specifically, treatment with the extracts led to a more than 2-fold increase in the biosynthesis of doxorubicin, apramycin, and neomycin. Conversely, the production of several other metabolites: clindamycin, bekanamycin, virginiamycin, dihydrostreptomycin, etamycin, rifamycin, streptomycin, and antimycin A (more than 2-fold lower). The affected metabolites belong to a diverse range of antibiotic classes, including macrolides (doxorubicin), aminoglycosides (neomycin, bekanamycin, and streptomycin), lincosamides (clindamycin), streptogramins (virginiamycin and etamycin), ansamycins (rifamycin), and others (antimycin A). This broad-spectrum effect suggests that the extract triggers a complex, large-scale reprogramming of secondary metabolism in *S. albulus*, diverting resources and regulatory control across multiple biosynthetic pathways.

## 4. Discussion

The production of secondary metabolites by *Streptomyces* species is highly regulated through complex and precise systems at the transcriptional level. Elicitors play a significant role in these regulations. These elicitors are typically categorized as chemical compounds (metals, rare earth elements, dimethyl sulfoxide, ethanol, and nanoparticles) [37,38,39,40,41,42,43,44] and environmental stressors (temperature shifts, pH shock, and dissolved oxygen levels) [45,46,47,48,49]. Recently, stress derived from heterogeneous microorganisms has been identified as a novel elicitor that intricately regulates the biosynthesis of secondary metabolites. The influence varies depending on the species of microorganisms. Generally, fungi secrete metabolic compounds that affect antibiotic synthesis in *Streptomyces*. For example, rimocidin production can be improved by adding broth and cells of *Saccharomyces cerevisiae* and *F. oxysporum f.* sp. *cucumerinum* due to increased transcription of genes [50]. Some fungi can generate elicitors that promote the biosynthesis of natamycin by *S. natalensis* HW-2, possibly due to an increased precursor supply through the regulation of amino acid metabolism [51,52,53]. However, heterogeneous *Streptomyces* influence the production of secondary metabolites through mycelia–mycelia physical interaction [54]. Interestingly, in this study, a heterogeneous *Streptomyces*, *S. gilvosporeus*, was also capable of secreting certain extract molecules to induce overproduction of ε-PL in *S. albulus,* similar to fungi [28], although they might operate through different physiological mechanisms.

In contrast to the effects of fungal extracts (e.g., *B. cinerea*) [28], the ε-PL-producing strain exhibits a distinct response to extracts from closely related *Actinomycetes* species (e.g., *S. gilvosporeus*) (Table S3). Specifically, (i) the variation in suitable solvents (butyl alcohol for *B. cinerea* and ethyl acetate for *S. gilvosporeus*) underscores the structural divergence of extract molecules derived from these two organisms; (ii) the extracts from *S. gilvosporeus* demonstrate a more potent inducing effect (3.42 ± 0.27 g/L ε-PL yield) compared to those from *B. cinerea* (1.88 ± 0.21 g/L ε-PL yield); (iii) the extracts from *S. gilvosporeus* lead to denser mycelial pellets, while the extracts from *B. cinerea* promote more robust and aggregated pellets, suggesting a potential physical confrontation between the ε-PL-producing strain and molds upon receiving the *B. cinerea* extracts; (iv) the *B. cinerea* extracts globally upregulate enzymes involved in ε-PL biosynthesis, while the *S. gilvosporeus* extracts specifically enhance the rate-limiting enzymes in the ε-PL biosynthetic pathways, indicating that the *S. gilvosporeus* extracts can induce more precise metabolic regulation in the ε-PL-producing strain; (v) the *S. gilvosporeus* extracts upregulate four phospholipase C genes and three phospholipase D genes, in contrast to the single phospholipase C gene upregulated by the *B. cinerea* extracts, demonstrating that the *S. gilvosporeus* extracts lead to stronger phospholipid hydrolysis, thereby increasing membrane fluidity and facilitating the transmembrane transport of nutrients, products, and extracts; and (vii) the *B. cinerea* extracts result in higher levels of ATP, NADH, and NADPH (and lower ADP, NAD^+^, and higher NADP^+^), whereas the *S. gilvosporeus* extracts induce lower ATP, NADH, and NADPH (and higher ADP, NAD^+^, and NADP^+^). Given the enhanced cell respiration of *S. albulus* IFO14147 observed under both *B. cinerea* and *S. gilvosporeus* extracts, the *S. gilvosporeus* extracts may indicate a greater demand for energy, which is likely utilized in ε-PL biosynthesis.

The coordinated physiological responses to *S. gilvosporeus* extract suggest a global reprogramming of metabolism in *S. albulus*. Enhanced expression and activity of key enzymes in glycolysis, the TCA cycle, and the pentose phosphate pathway indicate metabolic rerouting toward precursor supply and energy regeneration. The significant elevation of phosphoenolpyruvate carboxykinase, combined with increased intracellular glutamic acid and cysteine, supports improved carbon and nitrogen flux and potential oxidative stress adaptation. Notably, the reduction in aspartate and lysine pools likely reflects their enhanced consumption in ε-PL synthesis. These data support the hypothesis that the extract functions as a signaling molecule, triggering elevated fluxes in multiple metabolic nodes.

ε-PL is a secondary metabolite produced by *S. albulus*, known for its strong H^+^ absorption and antimicrobial activity [55]. Since it is not essential for growth, ε-PL likely serves a specific physiological role. Studies show that ε-PL synthesis increases under acidic pH conditions to counteract high extracellular H^+^ levels [13,14,15,16]. This research also identifies potential microbial signals from fungi or actinomycetes as a key factor influencing ε-PL production. These inducing effects are complex, depending on microbial groups and physiological changes. Compared to our previous study [28], this study reveals that actinomycete extracts have a stronger impact on ε-PL production than fungal extracts.

Despite the apparent complexity of physiological changes, a discernible pattern can be identified. In response to microbial invasions in a low-dimensional ecosystem, cells shift into a hyper-efficient state. Research on *E. coli* and *B. subtilis* has revealed that the regulation of intracellular Ca^2+^ homeostasis and the perception of extracellular signals mediated by Ca^2+^ are of crucial significance in the biosynthesis of secondary metabolites [56]. Analogously, a comparable regulatory mechanism might exist in *Streptomyces* species. In these organisms, fluctuations in intracellular Ca^2+^ levels could potentially modulate the reprogramming of secondary metabolic networks by targeting key nodes within metabolic pathways [52]. Additionally, reactive oxygen species (ROS) can modulate secondary metabolites synthesis by altering the availability of precursor molecules, increasing the supply for ε-PL production while concurrently decreasing it for other secondary metabolites [36]. Regulation of this state involves the elevation of intracellular secondary messengers, such as H_2_O_2_ and Ca^2+^, which signal via the quorum-sensing system. This signaling leads to the formation of aggregated colonies that enhance resistance to external threats and potentially trigger comprehensive physiological modifications via ROS- and Ca^2+^-dependent regulatory pathways.

The above studies reveal that the active synthesis of ε-PL aims to eliminate surrounding microorganisms to protect nutrients and space. This disclosure of the intrinsic driving force for ε-PL production offers a wide range of possibilities for developing more efficient strategies to increase ε-PL production through the co-cultivation of multiple microorganisms, which has significant theoretical and practical implications for industrial ε-PL production.

## 5. Conclusions

This study demonstrates that actinomycete extracts can markedly enhance ε-PL production in *Streptomyces albulus*. Integrated multi-omics and morphological analyses suggest that *S. gilvosporeus* extracts can trigger a distinct physiological shift involving metabolic reprogramming and stress-response activation. These findings underscore the regulatory potential of interspecies interactions and offer a promising ecological strategy for improving ε-PL biosynthesis in industrial applications.

## Figures and Tables

**Table 1:** Strains used in this study.

Strains Role	Strains	Origin
ε-PL-producing strain	*Streptomyces albulus* IFO14147	CICC 11022
Inducing strain (bacteria)	*Escherichia coli*	CICC 10389
	*Bacillus subtilis*	CICC 10002
	*Micrococcus luteus*	CICC 10269
	*Bacillus thuringiensis*	CICC 10061
	*Corynebacterium glutamicum*	CICC 20182
	*Pseudomonas aeruginosa*	CICC 10419
Inducing strain (fungi)	*Botrytis cinerea*	CGMCC 3.3790
	*Aspergillus niger*	CICC 40102
	*Monascus purpureus*	CICC 40942
	*Aspergillus oryzae*	CGMCC 3.7084
	*Penicillium chrysogenum*	CGMCC 3.15725
	*Saccharomyces cerevisiae*	CICC 1302
Inducing strain (actinomycetes)	*Streptomyces gilvosporeus*	ATCC 13326
	*Streptomyces coelicolor*	CGMCC 4.3587
	*Streptomyces lividans*	CGMCC 4.7169
	*Streptomyces diastatochromogenes*	CICC 11011
	*Streptomyces atratus*	CICC 11048
	*Streptomyces aquilus*	CICC 11055

**Table 2:** Comparison of gene transcription related to lipid catabolism between cultures of EG (experiment group, with *S. gilvosporeus* extract supplement) and CK (control group, without extract supplement).

Gene ID	Log_2_^(EG/CK)^	*p*-Value	Description
*N1H47_00700*	1.48759231	4.55 × 10^−4^	Phospholipase D
*N1H47_29515*	3.218336643	2.46 × 10^−12^	Phospholipase C
*N1H47_18160*	4.370954313	1.21 × 10^−15^	Phospholipase C
*N1H47_18130*	−0.115759649	0.819	Phospholipase D
*N1H47_33410*	−0.105715175	0.827	Phospholipase C
*N1H47_33420*	−0.28392415	0.584	Phospholipase C
*N1H47_39580*	−0.037235596	0.954	Phospholipase D

**Table 3:** Comparison of gene transcription related to energy metabolism between cultures of EG (experiment group, with *S. gilvosporeus* extract supplement) and CK (control group, without extract supplement).

Gene ID	Log_2_^(EG/CK)^	*p-*Value	Description
*N1H47_17980*	1.40	0.001	NADH-quinone oxidoreductase subunit D
*N1H47_17995*	1.36	0.001	NADH-quinone oxidoreductase subunit A
*N1H47_17970*	1.25	0.003	NADH-quinone oxidoreductase subunit F
*N1H47_17985*	1.20	0.004	NADH-quinone oxidoreductase subunit C
*N1H47_17990*	1.20	0.004	NADH-quinone oxidoreductase subunit B
*N1H47_17975*	1.15	0.006	NADH-quinone oxidoreductase subunit E
*N1H47_17960*	1.01	0.015	NADH-quinone oxidoreductase subunit H
*N1H47_26260*	0.24	0.561	F0F1 ATP synthase subunit beta
*N1H47_26255*	0.28	0.503	F0F1 ATP synthase subunit gamma
*N1H47_26240*	0.07	0.860	F0F1 ATP synthase subunit B
*N1H47_26245*	0.01	0.985	F0F1 ATP synthase subunit delta
*N1H47_26235*	0.11	0.789	F0F1 ATP synthase subunit C
*N1H47_26265*	0.38	0.363	F0F1 ATP synthase subunit epsilon
*N1H47_26230*	0.03	0.936	F0F1 ATP synthase subunit A

## Data Availability

The data presented in this study are openly available in [Streptomyces noursei strain IFO14147 chromosome, complete genome] at [https://www.ncbi.nlm.nih.gov/nuccore/NZ_CP104098.1] [NZ_CP104098.1]. Accessed on 30 June 2025.

## Supplementary Materials

The following supporting information can be downloaded at: https://www.mdpi.com/article/10.3390/microorganisms13081868/s1, Table S1: Relative fold change of compounds involved in ε-PL biosynthesis by *S. albulus* after *S. gilvosporeus* signal addition according to UPLC-ESI-MS analysis. Note: ns indicates no statistical significance (*p* ≥ 0.05); * indicates *p* < 0.05; ** indicates *p* < 0.01; *** indicates *p* < 0.001; **** indicates *p* < 0.0001. Table S2: Relative fold change of compounds involved in other secondary metabolites biosynthesis by *S. albulus* after *S. gilvosporeus* signal addition according to UPLC-ESI-MS analysis. Note: ns indicates no statistical significance (*p* ≥ 0.05); * indicates *p* < 0.05; ** indicates *p* < 0.01; *** indicates *p* < 0.001; **** indicates *p* < 0.0001. Table S3: Comparison of the physiological response under different interspecies signaling. Table S4: Primer pairs sequences for quantitative real-time PCR (qRT-PCR) assay.

## References

[1] Yamanaka K., Maruyama C., Takagi H., Hamano Y. (2008). ε-poly-L-lysine dispersity is controlled by a highly unusual nonribosomal peptide synthetase. Nat. Chem. Biol..

[2] Bhattacharya S., Dineshkumar R., Dhanarajan G., Sen R., Mishra S. (2017). Improvement of ε-polylysine production by marine bacterium *Bacillus licheniformis* using artificial neural network modeling and particle swarm optimization technique. Biochem. Eng. J..

[3] Dou Y., Routledge M.N., Gong Y., Godana E.A., Dhanasekaran S., Yang Q., Zhang X., Zhang H. (2021). Efficacy of epsilon-poly-L-lysine inhibition of postharvest blue mold in apples and potential mechanisms. Postharvest Biol. Technol..

[4] Jiao W., Liu X., Chen Q., Du Y., Li Y., Yue F., Dong X., Fu M. (2020). Epsilon-poly-L-lysine (ε-PL) exhibits antifungal activity in vivo and in vitro against *Botrytis cinerea* and the mechanism involved. Postharvest Biol. Technol..

[5] Li F., Wu S., Xu B. (2021). Preservation of stewed beef chunks by using ε-polylysine and tea polyphenols. LWT–Food Sci. Technol..

[6] Li W., Lv J., Dong T., Li X., Li X., Tan Z., Jia S. (2021). Effects of amino acids and overexpression of *dapA* gene on the production of ε-poly-L-lysine by *Streptomyces diastatochromogenes* strains. Curr. Microbiol..

[7] Tsukatani T., Kuroda R., Kawaguchi T. (2022). Screening biofilm eradication activity of ethanol extracts from foodstuffs: Potent biofilm eradication activity of glabridin, a major flavonoid from licorice (*Glycyrrhiza glabra*), alone and in combination with ɛ-poly-L-lysine. World J. Microbiol. Biotechnol..

[8] Chen S., Huang S., Li Y., Zhou C. (2021). Recent advances in epsilon-poly-L-lysine and L-lysine-based dendrimer synthesis, modification, and biomedical applications. Front. Chem..

[9] Zhao Y.Z., Shen B.X., Li X.Z., Tong M.Q., Xue P.P., Chen R., Yao Q., Chen B., Xiao J., Xu H.L. (2020). Tumor cellular membrane camouflaged liposomes as a non-invasive vehicle for genes: Specific targeting toward homologous gliomas and traversing the blood–brain barrier. Nanoscale.

[10] Li S., Chen N., Li Y., Li X., Zhan Q., Ban J., Zhao J., Hou X., Yuan X. (2020). Metal-crosslinked ε-poly-L-lysine tissue adhesives with high adhesive performance: Inspiration from mussel adhesive environment. Int. J. Biol. Macromol..

[11] Hyon W., Shibata S., Ozaki E., Fujimura M., Hyon S.H., Matsumura K. (2022). Elucidating the degradation mechanism of a self-degradable dextran-based medical adhesive. Carbohydr. Polym..

[12] Shen S., Liu X., Huang J., Sun Y., Liu B., Song W., Meng L., Du M., Feng Q. (2024). Efficacy of a mouthwash containing ε-poly-L-lysine, funme peptides, and domiphen in reducing halitosis and supragingival plaque: A randomized clinical trial. BMC Oral Health.

[13] Hiraki J., Hatakeyama M., Morita H., Izumi Y. (1998). Improved ε-poly-L-lysine production of an S-(2-aminoethyl)-L-cysteine resistant mutant of *Streptomyces albulus*. Seibutsu Kogaku Kaishi.

[14] Liu Y.J., Wang K.F., Pan L., Chen X.S. (2022). Improved production of ε-poly-L-lysine in *Streptomyces albulus* using genome shuffling and its high-yield mechanism analysis. Front. Microbiol..

[15] Wang L., Li S., Zhao J., Liu Y., Chen X., Tang L., Mao Z. (2019). Efficiently activated ε-poly-L-lysine production by multiple antibiotic-resistance mutations and acidic pH shock optimization in *Streptomyces albulus*. MicrobiologyOpen.

[16] Wang A., Tian W., Cheng L., Xu Y., Wang X., Qin J., Yu B. (2020). Enhanced ε-poly-L-lysine production by the synergistic effect of ε-poly-L-lysine synthetase overexpression and citrate in *Streptomyces albulus*. Front. Bioeng. Biotechnol..

[17] Wang L., Yang H., Wu M., Zhang H., Zhang J., Chen X. (2024). Enhanced ε-poly-L-lysine production in *Streptomyces albulus* through multi-omics-guided metabolic engineering. Biomolecules.

[18] Kahar P., Iwata T., Hiraki J., Park E.Y., Okabe M. (2001). Enhancement of ε-poly-L-lysine production by *Streptomyces albulus* strain 410 using pH control. J. Biosci. Bioeng..

[19] Jia S.R., Wang G.L., Sun Y.F., Tan Z.L. Improvement of ε-poly-L-lysine production by *Streptomyces albulus* TUST2 employing a feeding strategy. Proceedings of the International Conference on Bioinformatics and Biomedical Engineering (iCBBE).

[20] Xu Z., Bo F., Xia J., Sun Z., Li S., Feng X., Xu H. (2015). Effects of oxygen-vectors on the synthesis of epsilon-poly-lysine and the metabolic characterization of *Streptomyces albulus* PD-1. Biochem. Eng. J..

[21] Xia J., Xu Z., Xu H., Feng X., Bo F. (2014). The regulatory effect of citric acid on the co-production of poly(ε-lysine) and poly(L-diaminopropionic acid) in *Streptomyces albulus* PD-1. Bioproc. Biosyst. Eng..

[22] Liu S., Wu Q., Zhang J., Mo S. (2011). Production of ε-poly-L-lysine by *Streptomyces* sp. using resin-based, in situ product removal. Biotechnol. Lett..

[23] Ren X.D., Chen X.S., Zeng X., Wang L., Tang L., Mao Z.G. (2015). Acidic pH shock induced overproduction of ε-poly-L-lysine in fed-batch fermentation by *Streptomyces* sp. M-Z18 from agro-industrial by-products. Bioproc. Biosyst. Eng..

[24] Pan L., Chen X.S., Liu M.M., Liu Y.J., Mao Z.G. (2017). Efficient production of ε-poly-L-lysine from glucose by two-stage fermentation using pH shock strategy. Process Biochem..

[25] Pan L., Chen X.S., Wang K.F., Mao Z.G. (2019). Understanding of high ε-poly-L-lysine production by *Streptomyces albulus* using pH shock strategy in the level of transcriptomics. J. Ind. Microbiol. Biot..

[26] Pan L., Chen X.S., Wang K.F., Mao Z.G. (2019). A temporal transcriptomic dynamics study reveals the reason of enhanced ε-poly-L-lysine production in *Streptomyces albulus* M-Z18 by pH shock. Process Biochem..

[27] Pan L., Chen X.S., Wang K.F., Mao Z.G. (2020). Mechanisms of response to pH shock in microbial fermentation. Bioproc. Biosyst. Eng..

[28] Zeng X., Zhang C., Yue C., Su Z., Tai B., Tang H., Zeng H., Xin B., Zhu M. (2022). Transcriptome and metabolome analysis revealing the improved ε-poly-L-lysine production induced by a microbial call from *Botrytis cinerea*. Appl. Environ. Microbiol..

[29] Zeng X., Chen X.S., Gao Y., Ren X.D., Wang L., Mao Z.G. (2015). Continuously high reactive oxygen species generation decreased the specific ε-poly-L-lysine formation rate in fed-batch fermentation using glucose and glycerol as a mixed carbon source. Process Biochem..

[30] Zeng X., Miao W.Y., Wen B.B., Mao Z.G., Zhu M.Z., Chen X.S. (2019). Transcriptional study of the enhanced ε-poly-L-lysine productivity in culture using glucose and glycerol as a mixed carbon source. Bioproc. Biosyst. Eng..

[31] Zhang C., Zhang Z., Cheng Y., Ni N., Tong S., Da W., Liu C., Diao Q., Chen Z., Xin B. (2024). Transcriptional analysis revealing the improvement of ε-poly-L-lysine production from intracellular ROS elevation after *Botrytis cinerea* induction. J. Fungi.

[32] Zeng X., Chen X.S., Ren X.D., Liu Q.R., Wang L., Sun Q.X., Tang L., Mao Z.G. (2014). Insights into the role of glucose and glycerol as a mixed carbon source in the improvement of ε-poly-L-lysine productivity. Appl. Biochem. Biotech..

[33] Herrera A., Gómez M., Packard T.T., Reglero P., Blanco E., Barberá-Cebrián C. (2014). Potential respiration estimated by electron transport system activity in deep-sea suprabenthic crustaceans off Balearic Islands (Western Mediterranean). J. Marine Syst..

[34] Schalk P.H. (1988). Respiratory electron transport system (ETS) activities in zooplankton and micronekton of the Indo-Pacific region. Mar. Ecol. Prog. Ser..

[35] Cammen L.M., Corwin S., Christensen J. (1990). Electron transport system (ETS) activity as a measure of benthic macrofaunal metabolism. Mar. Ecol. Prog. Ser..

[36] Yue C., Su Z., Tai B., Tang H., Da W., Xu H., Zeng H., Xin B., Zeng X. (2023). Physiological analysis of the improved ε-poly-L-lysine production induced by reactive oxygen species. Appl. Microbiol. Biotechnol..

[37] Wang L., Li Y., Li Y. (2019). Metal ions driven production, characterization and bioactivity of extracellular melanin from *Streptomyces sp.* ZL-24. Int. J. Biol. Macromol..

[38] Kawai K., Wang G., Okamoto S., Ochi K. (2007). The rare earth, scandium, causes antibiotic overproduction in *Streptomyces* spp.. FEMS Microbiol. Lett..

[39] Wang C., Huang D., Liang S. (2018). Identification and metabolomic analysis of chemical elicitors for tacrolimus accumulation in *Streptomyces tsukubaensis*. Appl. Microbiol. Biot..

[40] Wang C., Wang J., Yuan J., Yuan J., Jiang L., Jiang X., Yang B., Zhao G., Liu B., Huang D. (2019). Generation of *Streptomyces hygroscopicus* cell factories with enhanced ascomycin production by combined elicitation and pathway-engineering strategies. Biotechnol. Bioeng..

[41] Bhatia S.K., Lee B.R., Sathiyanarayanan G., Song H.S., Kim J., Jeon J.M., Kim J.H., Park S.H., Yu J.H., Park K. (2016). Medium engineering for enhanced production of undecylprodigiosin antibiotic in *Streptomyces coelicolor* using oil palm biomass hydrolysate as a carbon source. Bioresour. Technol..

[42] Sekurova O.N., Zhang J., Kristiansen K.A., Zotchev S.B. (2016). Activation of chloramphenicol biosynthesis in *Streptomyces venezuelae* ATCC 10712 by ethanol shock: Insights from the promoter fusion studies. Microb. Cell Fact..

[43] Liu X., Tang J., Wang L., Liu R. (2019). Mechanism of CuO nano-particles on stimulating production of actinorhodin in *Streptomyces coelicolor* by transcriptional analysis. Sci. Rep..

[44] Liu X., Tang J., Wang L., Tang J., Wang L., Giesy J.P. (2019). Al_2_O_3_ nanoparticles promote secretion of antibiotics in *Streptomyces coelicolor* by regulating gene expression through the nano effect. Chemosphere.

[45] Bursy J., Kuhlmann A.U., Pittelkow M., Hartmann H., Jebbar M., Pierik A.J., Bremer E. (2008). Synthesis and uptake of the compatible solutes ectoine and 5-hydroxyectoine by *Streptomyces coelicolor* A3(2) in response to salt and heat stresses. Appl. Environ. Microbiol..

[46] Bucca G., Pothi R., Hesketh A., Möller-Levet C., Hodgson D.A., Laing E.E., Stewart G.R., Smith C.P. (2018). Translational control plays an important role in the adaptive heatshock response of *Streptomyces coelicolor*. Nucleic Acids Res..

[47] Mo S., Kim J.H., Oh C.H. (2013). Different effects of acidic pH shock on the prodiginine production in *Streptomyces coelicolor* M511 and SJM1 mutants. J. Microbiol. Biotechn..

[48] Jiang J., Sun Y.F., Tang X., He C.N., Shao Y.L., Tang Y., Zhou W.W. (2018). Alkaline pH shock enhanced production of validamycin A in fermentation of *Streptomyces hygroscopicus*. Bioresource Technol..

[49] Kaiser D., Onken U., Sattler I., Zeeck A. (1994). Influence of increased dissolved oxygen concentration on the formation of secondary metabolites by manumycin-producing *Streptomyces parvulus*. Appl. Microbiol. Biot..

[50] Song Z., Ma Z., Bechthold A., Yu X. (2020). Effects of addition of elicitors on rimocidin biosynthesis in *Streptomyces rimosus* M527. Appl. Microbiol. Biot..

[51] Wang D., Yuan J., Gu S., Shi Q. (2013). Influence of fungal elicitors on biosynthesis of natamycin by *Streptomyces natalensis* HW-2. Appl. Microbiol. Biot..

[52] Wang D., Wei L., Zhang Y., Zhang M., Gu S. (2017). Physicochemical and microbial responses of *Streptomyces natalensis* HW-2 to fungal elicitor. Appl. Microbiol. Biotechnol..

[53] Shen W., Wang D., Wei L., Zhang Y. (2020). Fungal elicitor-induced transcriptional changes of genes related to branched-chain amino acid metabolism in *Streptomyces natalensis* HW-2. Appl. Microbiol. Biotechnol..

[54] Zong G., Fu J., Zhang P., Zhang W., Xu Y., Cao G., Zhang R. (2022). Use of elicitors to enhance or activate the antibiotic production in *Streptomyces*. Crit. Rev. Biotechnol..

[55] Wang L., Zhang C.Y., Zhang J.H., Rao Z.M., Xu X.M., Mao Z.G., Chen X.S. (2021). Epsilon-poly-L-lysine: Recent advances in biomanufacturing and applications. Front. Bioeng. Biotechnol..

[56] Murphy T.M., Nilsson A.Y., Roy I., Harrop A., Dixon K., Keshavarz T. (2011). Enhanced intracellular Ca^2+^ concentrations in *Escherichia coli* and *Bacillus subtilis* after addition of oligosaccharide elicitors. Biotechnol. Lett..

