# Supplementary material for: Physiological Insights into Enhanced Epsilon-Poly-l-Lysine Production Induced by Extract Supplement from Heterogeneous Streptomyces Strain
Source: Microorganisms. 2025 Aug 10;13(8):1868. doi: 10.3390/microorganisms13081868 (PMC12388667; doi:10.3390/microorganisms13081868)
Supplement: Supplementary file 1 [file microorganisms-13-01868-s001.zip › microorganisms-3781640-supplementary.pdf]

Table S1 Relative fold change of compounds involved in ε-PL biosynthesis by *S. albulus* after *S. gilvosporeus* signal addition according to UPLC-ESI-MS analysis.

ID	t_R (min)	Measured mass	Formula	Name	Fold change		
					CK/CK	EG/CK	
				**Embden-Meyerhof pathway**			
1	2.8767	180.0634	$C_6H_{12}O_6$	D-Glucose	1.00	1.18	ns
2	0.7333	260.0297	$C_6H_{13}O_9P$	Glucose 6-phosphate	1.00	1.78	****
3	11.3750	339.9960	$C_6H_{14}O_{12}P_2$	Fructose 1,6-bisphosphate	1.00	0.80	*
4	4.4067	169.9980	$C_3H_7O_6P$	D-Glyceraldehyde 3-phosphate	1.00	2.99	***
				**Tricarboxylic acid cycle**			
5	0.6383	192.0270	$C_6H_8O_7$	Citric acid	1.00	0.99	ns
6	5.6700	132.0059	$C_4H_4O_5$	Oxalacetic acid	1.00	1.39	ns
7	0.7300	118.0266	$C_4H_6O_4$	Succinic acid	1.00	1.06	ns
8	0.7450	116.0110	$C_4H_4O_4$	Fumaric acid	1.00	0.89	ns
				**Pentose phosphate pathway**			
9	0.9033	178.0477	$C_6H_{10}O_6$	Gluconolactone	1.00	1.01	ns
10	1.5900	276.0246	$C_6H1_3O_{10}P$	6-Phosphogluconic acid	1.00	0.84	ns
11	0.7467	230.0192	$C_5H_{11}O_8P$	D-Ribulose 5-phosphate	1.00	1.05	ns
				**Diaminopimelic acid pathway**			
12	0.8150	133.0375	$C_4H_7NO_4$	L-Aspartic acid	1.00	0.92	***
13	2.3167	171.0532	$C_7H_9NO_4$	Tetrahydrodipicolinate	1.00	0.98	ns
14	0.8350	190.0954	$C_7H_{14}N_2O_4$	Diaminopimelic acid	1.00	1.54	****
				**Cofactors**			
15	9.8517	506.9957	$C_{10}H_{16}N_5O_{13}P_3$	ATP	1.00	0.32	****
16	5.2050	427.0294	$C_{10}H_{15}N_5O_{10}P_2$	ADP	1.00	1.93	****
17	1.0300	347.0631	$C_{10}H_{14}N_5O_7P$	AMP	1.00	1.01	ns
18	7.4400	664.1169	$C_{21}H_{28}N_7O_{14}P_2$	NAD	1.00	1.24	ns
19	8.0117	665.1248	$C_{21}H_{29}N_7O_{14}P_2$	NADH	1.00	0.35	**
20	4.8533	744.0833	$C_{21}H_{29}N_7O_{17}P_3$	NADP	1.00	12.06	****
21	9.5500	745.0911	$C_{21}H_{30}N_7O_{17}P_3$	NADPH	1.00	0.19	**
				**Amino acids**			
22	0.7217	146.1055	$C_6H_{14}N_2O_2$	L-Lysine	1.00	1.05	ns
23	0.7383	155.0695	$C_6H_9N_3O_2$	L-Histidine	1.00	1.10	ns
24	0.7350	174.1117	$C_6H_{14}N_4O_2$	L-Arginine	1.00	0.96	ns
25	0.7200	146.0691	$C_5H_{10}N_2O_3$	L-Glutamine	1.00	0.52	*
26	0.8167	119.0582	$C_4H_9NO_3$	L-Threonine	1.00	1.55	ns
27	1.8167	240.0238	$C_6H_{12}N_2O_4S_2$	L-Cystine	1.00	5.90	****
28	0.8017	105.0426	$C_3H_7NO_3$	L-Serine	1.00	1.12	ns
29	0.8450	115.0633	$C_5H_9NO_2$	L-Proline	1.00	0.96	ns
30	0.8217	147.0532	$C_5H_9NO_4$	L-Glutamic acid	1.00	1.50	****
31	1.1433	149.051	$C_5H_{11}NO_2S$	L-Methionine	1.00	1.24	****
32	6.8883	131.0946	$C_6H_{13}NO_2$	L-Isoleucine	1.00	0.78	ns
33	3.2750	131.0946	$C_6H_{13}NO_2$	L-Leucine	1.00	1.36	ns
34	0.9183	117.079	$C_5H_{11}NO_2$	L-Valine	1.00	0.97	ns

35	1.7567	165.079	$C_9H_{11}NO_2$	L-Phenylalanine	1.00	1.19	***
36	1.1783	181.0739	$C_9H_{11}NO_3$	L-Tyrosine	1.00	0.98	ns
37	3.5633	204.0899	$C_{11}H_{12}N_2O_2$	L-Tryptophan	1.00	0.92	ns
38	0.8050	132.0535	$C_4H_8N_2O_3$	L-Asparagine	1.00	0.50	*

Table S2 Relative fold change of compounds involved in other secondary metabolites biosynthesis by *S. albulus* after *S. gilvosporeus* signal addition according to UPLC-ESI-MS analysis.

**ID**	**t_R (min)**	**Measured mass**	**Formula**	**Name**	**Fold change**		
					**CK/CK**	**EG/CK**	**Significance**
1	1.1633	291.0967	$C_{12}H_{13}N_{5}O_{4}$	Toyocamycin	1.00	1.57	***
2	10.6767	557.1897	$C_{28}H_{31}NO_{11}$	Rhodomycin D	1.00	0.67	*
3	5.8983	265.1314	$C_{14}H_{19}NO_{4}$	Anisomycin	1.00	1.48	**
4	5.0217	334.1277	$C_{15}H_{18}N_{4}O_{5}$	Mitomycin	1.00	0.80	ns
5	4.6567	424.1799	$C_{18}H_{33}C_{l}N_{2}O_{5}S$	Clindamycin	1.00	0.09	****
6	3.6050	581.5935	$C_{18}H_{37}N_{5}O_{10}.H_{2}SO_{4}$	Bekanamycin sulfate	1.00	0.35	**
7	4.4000	499.1842	$C_{26}H_{29}NO_{9}$	13-Deoxycarminomycin	1.00	1.27	*
8	0.7467	543.1741	$C_{27}H_{29}NO_{11}$	Doxorubicin	1.00	3.07	***
9	5.0667	379.1591	$C_{14}H_{25}N_{3}O_{9}$	Kasugamycin	1.00	0.97	ns
10	4.0100	823.3541	$C_{43}H_{49}N_{7}O_{10}$	Virginiamycin	1.00	0.48	**
11	3.6467	332.1584	$C_{14}H_{24}N_{2}O_{7}$	Spectinomycin	1.00	0.66	***
12	1.9517	335.1117	$C_{15}H_{17}N_{3}O_{6}$	Mitomycin B	1.00	0.98	ns
13	3.6983	527.2326	$C_{20}H_{37}N_{3}O_{13}$	Hygromycin B	1.00	0.87	ns
15	6.1550	811.3415	$C_{42}H_{53}NO_{15}$	Aclacinomycin A	1.00	1.47	ns
16	2.9950	484.2381	$C_{18}H_{36}N_{4}O_{11}$	Kanamycin	1.00	0.93	ns
17	7.4567	540.3451	$C_{33}H_{48}O_{6}$	Leptomycin B	1.00	0.91	ns
18	3.9117	539.2802	$C_{21}H_{41}N_{5}O_{11}$	Apramycin sulfate	1.00	3.85	****
19	4.7633	542.7034	$C_{32}H_{46}O_{7}$	Milbemycin A4	1.00	1.09	ns
20	3.6367	539.2803	$C_{21}H_{41}N_{5}O_{11}$	Apramycin	1.00	0.77	*
21	1.4600	560.2734	$C_{29}H_{40}N_{2}O_{9}$	Geldanamycin	1.00	0.95	ns
22	3.6333	583.2813	$C_{21}H_{41}N_{7}O_{12}$	Dihydrostreptomycin	1.00	0.29	****
23	4.1867	515.3029	$C_{25}H_{45}N_{3}O_{6}S$	Carmamycin A	1.00	0.91	ns
24	4.3067	615.2963	$C_{23}H_{45}N_{5}O_{14}$	Paromomycin	1.00	1.83	*
25	4.7983	614.3123	$C_{23}H_{46}N_{6}O_{13}$	Neomycin	1.00	2.72	****
26	4.7200	733.4612	$C_{37}H_{67}NO_{13}$	Erythromycin	1.00	1.25	ns
27	6.5567	772.4738	$C_{42}H_{69}NaO_{11}$	Salinomycin, Sodium	1.00	0.59	*
28	3.6450	878.4538	$C_{44}H_{62}N_{8}O_{11}$	Etamycin	1.00	0.44	**
29	3.6383	858.4474	$C_{40}H_{66}N_{4}O_{16}$	Tunicamycin X	1.00	0.95	ns
30	6.2250	523.2682	$C_{29}H_{37}N_{3}O_{6}$	Calcimycin	1.00	1.02	ns
31	2.1383	483.254	$C_{18}H_{37}N_{5}O_{10}$	Kanamycin B	1.00	0.71	ns
32	3.9283	841.4460	$C_{42}H_{67}NO_{16}$	Carbomycin	1.00	0.65	ns
33	6.6250	471.2230	$C_{22}H_{29}N_{7}O_{5}$	Puromycin	1.00	1.34	***
34	3.5617	406.2138	$C_{18}H_{34}N_{2}O_{6}S$	Lincomycin	1.00	1.55	ns
35	5.6800	697.3098	$C_{37}H_{47}NO_{12}$	Rifamycin	1.00	0.49	*
36	6.6967	861.5086	$C_{43}H_{75}NO_{16}$	Erythromycin ethylsuccinate	1.00	0.91	ns
37	7.4417	581.2657	$C_{21}H_{39}N_{7}O_{12}$	Streptomycin	1.00	0.45	**
38	1.1867	548.2734	$C_{28}H_{40}N_{2}O_{9}$	Antimycin A	1.00	0.15	***
39	7.0233	467.2591	$C_{18}H_{37}N_{5}O_{9}$	Tobramycin	1.00	1.47	**
40	8.3617	748.5085	$C_{38}H_{72}N_{2}O_{12}$	Azithromycin	1.00	1.52	*

41	10.2667	836.5246	$C_{41}H_{76}N_2O_{15}$	Roxithromycin	1.00	1.59	*

Table S3 Comparison of the physiological response under different interspecies signaling.

Performances after the interspecies signaling	*Botrytis cinerea* (Fungi)	*Streptomyces gilvosporeus* (Actinomycetes)
Suitable solvent for the signal molecules	Butyl alcohol	Ethyl acetate
ε-PL production in flasks	1.88±0.21 g/L	3.42±0.27 g/L
Transcriptional regulators involved	HrdD, LysR and quorum sensing systems	SenX3, RegX3, GntR, TetR, LysR, LuxR and quorum sensing systems
Mycelial morphology	Aggregating mycelial pellets with stronger mycelia	Denser mycelial pellets with normal mycelia
Metabolic enhancement for ε-PL biosynthesis	Global improvement in every enzymes	Specific improvement in rate-limiting enzymes
Upregulated genes in phospholipid hydrolysis	1 gene for Phospholipase C	4 genes for Phospholipase C<br>3 genes for Phospholipase D
Important intracellular cofactors' pools	Higher concentrations of ATP, ADP, NADH, NAD^+, NADPH, and lower concentrations of AMP, $NADP^+$	Higher concentrations of ADP, NAD^+, $NADP^+$, and lower concentrations of ATP, NADH, NADPH

Table S4 Primer pairs sequences for quantitative real-time PCR (qRT-PCR) assay.

Target gene	Primer name	Primer sequence (5’-3’)
16s rRNA	16s rRNA-forward	CAACGCGAAGAACCTTACCA
	16s rRNA -reverse	AGTCCCCATCACCCCGA
N1H47_11860	N1H47_11860 -forward	CAAGGAGACGCTCGCCAAG
	N1H47_11860 -reverse	GGAATGCCGTACTCCGTGAAG
N1H47_11275	N1H47_11275 -forward	GCGTCCACACCAAGGTCATC
	N1H47_11275 -reverse	GGTCGTCCTGGTCCTTCTCC
N1H47_14640	N1H47_14640 -forward	GGTGCTGGAGATGCTGGAAG
	N1H47_14640 -reverse	GCCGCCGCCTTGATGATC
N1H47_21215	N1H47_21215 -forward	CCCAGGACAAGAAGGACATCAC
	N1H47_21215 -reverse	GCAGACATCGGCGTTCAGG
N1H47_34205	N1H47_34205 -forward	GACGCCTGGATCATCTTCACC
	N1H47_34205 -reverse	GGCTGGTCCTGGCAGAACA
